# Development, design and pilot evaluation of a nurse practitioner led outpatient post-diagnostic clinic for people with dementia and their care partners: a brief report

**DOI:** 10.3389/frdem.2026.1843734

**Published:** 2026-07-22

**Authors:** Amy Montgomery, Wyatt Hampstead, Luke Molloy, Rebekkah Middleton, Nathan M. D’Cunha, Kelly Marriott-Statham

**Affiliations:** 1 Improving Palliative, Aged and Chronic Care through Clinical Research and Translation (IMPACCT), University of Technology Sydney, Sydney, NSW, Australia; 2 Faculty of Health, University of Wollongong, Wollongong, NSW, Australia; 3 Medicine in Motion Health Group, Vorrimal, NSW, Australia; 4 Centre for Ageing Research and Translation (CARAT), University of Canberra, Canberra, ACT, Australia

**Keywords:** dementia, memory clinic, non-pharmacological, nurse practitioner, post-diagnosis care, post-diagnostic support

## Abstract

**Background:**

The number of people with dementia is rising and need for access to post-diagnostic support involving non-pharmacological treatments is in demand. Non-pharmacological treatments cover a broad range of intervention categories, such as cognitive stimulation, art therapy and physical exercise.

**Objective:**

The objective of this brief report is to examine the development and design of a nurse practitioner-led post-diagnostic clinic, and assess its impact on participants cognition, psychological wellbeing and quality of life.

**Methods:**

The clinic was located at the University of Wollongong, a regional university in New South Wales, Australia. Participants were eligible if they had a clinical-confirmed diagnosis of dementia, living in the community (not residential aged care). Participants were invited to attend the clinic weekly, for 1–2 h, for up to 12-months and had the flexibility to choose their preferred activities each session.

**Results:**

A total of 16 people with dementia attended the clinic between September 2022 and June 2024. A total of eight out the 16 consented to participate in the study. The findings suggest that participants maintained their cognition, psychological wellbeing, and quality of life over the 12-month evaluation period, highlighting the clinic’s potential role in support stability in these domains.

**Conclusion:**

The strength of the clinic model is the unique and individualised way in which people with dementia were enabled to have choice in the activities and sessions they engaged with, reflecting person-centred practice values. This report provides structural guidance to develop and implement person-centred models of post-diagnostic care for dementia.

**Clinical trial registration:**

Identifier (ACTRN12625000014459).

## Introduction

Globally, the number of people with dementia is rising and with this the need for access to supportive post-diagnostic care. In Australia, over 433,300 people live with dementia, and with projections to increase to 812,500 by 2054 (Dementia Australia, 2025). Establishing new and effective support programs for people with dementia involving non-pharmacological treatments is paramount to reduce the impact of the condition and improve wellbeing and quality of life.

Non-pharmacological treatments, such as cognitive stimulation, art therapy, and physical exercise, have potential for meaningful impact on cognition, wellbeing, and quality of life for people with dementia (Sikkes et al., 2021). Cognitive stimulation involves activities and discussions to enhance cognitive and social function, and show short-term benefits for people with mild-to-moderate dementia, with limited evidence on the longer-term cognitive stimulation programs (>10 weeks) (Woods et al., 2023). Art therapy, which uses creativity to support cognitive, emotional, and interpersonal skills, improves wellbeing and quality of life but evidence is limited in dementia care (Deshmukh et al., 2018). Physical exercise has a growing evidence base in effectively slowing cognitive decline in people with dementia (Huang et al., 2022).

In Australia there is a need for further research into post-diagnostic dementia care models that incorporate multiple non-pharmacological treatments to ensure they are feasible and effective. Similar models of post-diagnostic support have been implemented internationally, including the Meeting Centres Support Programme and Cognitive Stimulation Therapy (CST) groups, which provide structured psychosocial support for people with dementia and their care partners, often delivered by care staff, registered nurses and allied health professionals (Dröes et al., 2004; Saragih et al., 2022; Spector et al., 2003, 2010; Szcześniak et al., 2021). Nurse practitioner (NP)-led clinics have been proposed as an innovative approach to care to improve access to post-diagnostic dementia care and support (Luck & Doucet, 2021). In the Australian context, NPs are advanced practice clinicians with extended scope, including autonomous assessment, diagnosis, prescribing and referral, positioning them well to lead integrated models of care. NP-led clinics for people with dementia are often multidisciplinary and include assessment, diagnosis, care planning, treatment (including prescribing), and referral to services (Luck & Doucet, 2021). However, the evidence base for these models is limited. To address the post-diagnostic needs of people with dementia and their care partners, a NP-led outpatient post-diagnostic clinic incorporating non-pharmacological treatments (cognitive stimulation, art therapy, and physical exercise) was developed. This brief report will focus on the development, design and pilot evaluation of the clinic.

## Aim

This brief report is part of a larger program evaluating an NP-led outpatient post-diagnostic clinic model and its impact on cognition, psychological wellbeing, and quality of life for people with dementia and their care partners. Outcomes from the care partners have been reported elsewhere (Beattie et al., 2025). The objectives of this brief report are to:

- Discuss the development and design of the NP-led post-diagnostic clinic for people with dementia and their care partners;
- Report on the pilot evaluation findings on the effect of the clinic on the cognition, psychological wellbeing and quality of life for people with dementia; and
- Present illustrative case vignettes to contextualise clinical application of the model.

## Methods

This study used a pilot study design. The study was registered on the Australian New Zealand Clinical Trials Registry (ACTRN12625000014459). The NP-led post-diagnostic clinic was located at the University of Wollongong, in regional New South Wales, Australia. This brief report presents data from the clinic opening in September 2022 to June 2024 and is structured in accordance with the Template for Intervention Description and Replication (TIDieR) checklist, to ensure comprehensive and transparent reporting (Hoffmann et al., 2014).

### Design of the clinic

The clinic was developed using a consultative design approach led by a NP (A. M), informed by inputs from key stakeholders, including academics, healthcare practitioners and people with lived experience of dementia. Two 60-min design consultation sessions were conducted via Zoom; one with healthcare practitioners [exercise physiologists (*n =* 2), aged care clinical nurse consultants (*n =* 2), aged care provider managers (*n =* 2) and geriatrician (*n =* 1)]; and one with people with lived experience of dementia (*n =* 6). Participants were purposively identified through existing clinical and research networks to ensure local representation of relevant clinical disciplines and lived experience perspectives. Stakeholders were invited to provide input on the structure, interventions and delivery of the clinic, including the selection and feasibility of non-pharmacological activities, and considerations for person-centred care. Feedback from these sessions informed the overall design and refinement of the clinic model.

The clinic design was built around an NP-led model, drawing on the advanced practice scope of the NP, including comprehensive assessment, diagnostic reasoning, prescribing, care coordination and referral. This enabled the integration of medical and psychosocial care within a single outpatient model, with an emphasis on individualised, goal-directed care. Each participant attended an initial appointment with the NP to gather baseline information, including medical history, occupation, hobbies, social activities, functional ability, and specific areas of concern. During this appointment the NP introduced the various activities available in the clinic. The information collected helped inform the design and modification of activities to suit each person’s goals, needs and preferences.

Participants were invited to attend the clinic weekly, for 1–2 h, for up to 12-months. Participants chose how often they attended during the 12-month period, and they chose their preferred activities each time they attended. The duration of the program was 12-months.

### Intervention development: individualised interventions

The design of the interventions offered were based on evidence-based principles, incorporating cognitive stimulation (Woods et al., 2023), art therapy (Deshmukh et al., 2018), and exercise interventions (Huang et al., 2022) to support people with dementia. Importantly, the design emphasised giving participants choice to engage in activities personally relevant and meaningful, and aligned with their personal goals.

**Figure 1:**
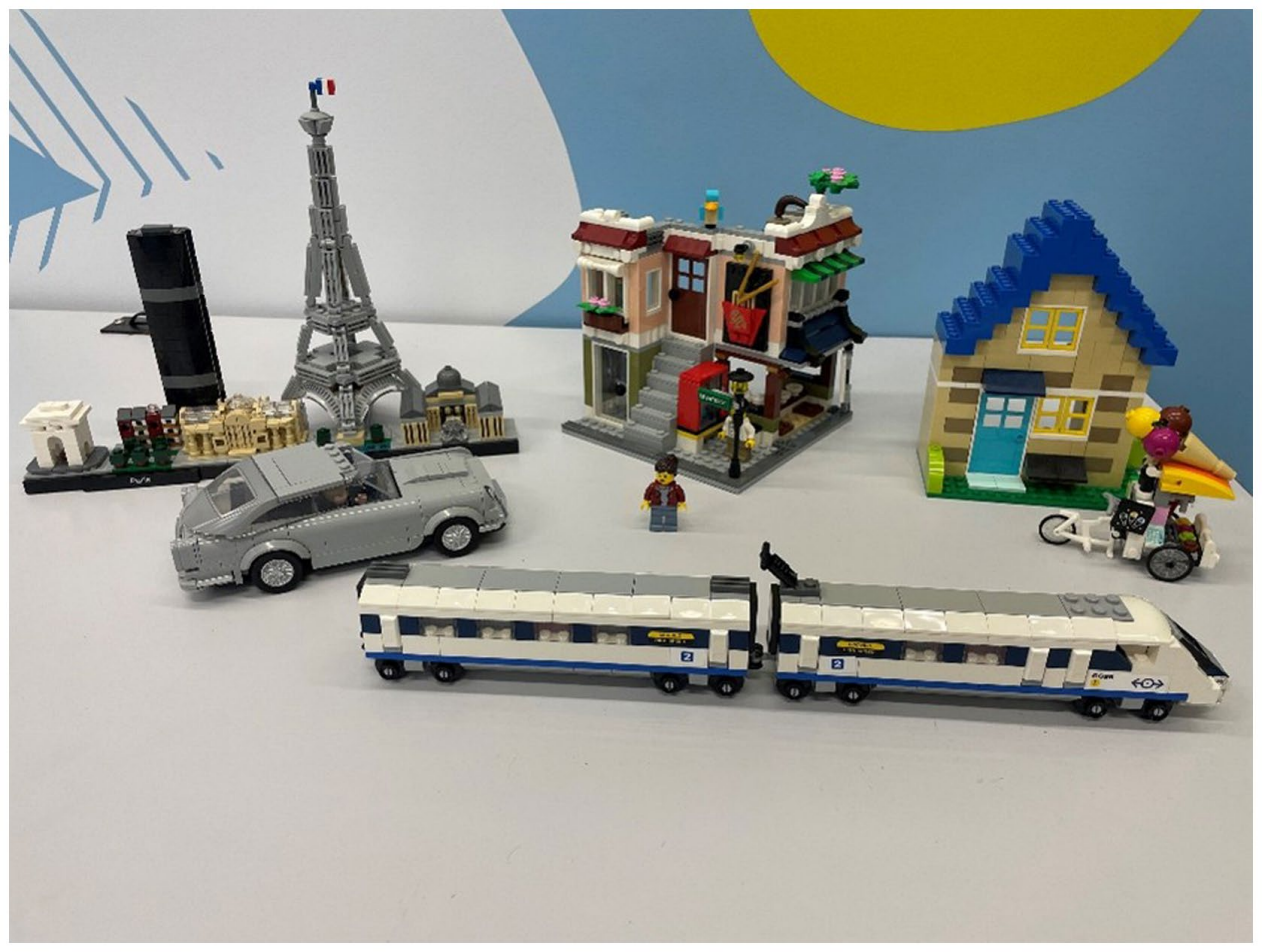
An example of LEGO^®^ models created by study participants during cognitive stimulation sessions.

**Figure 2:**
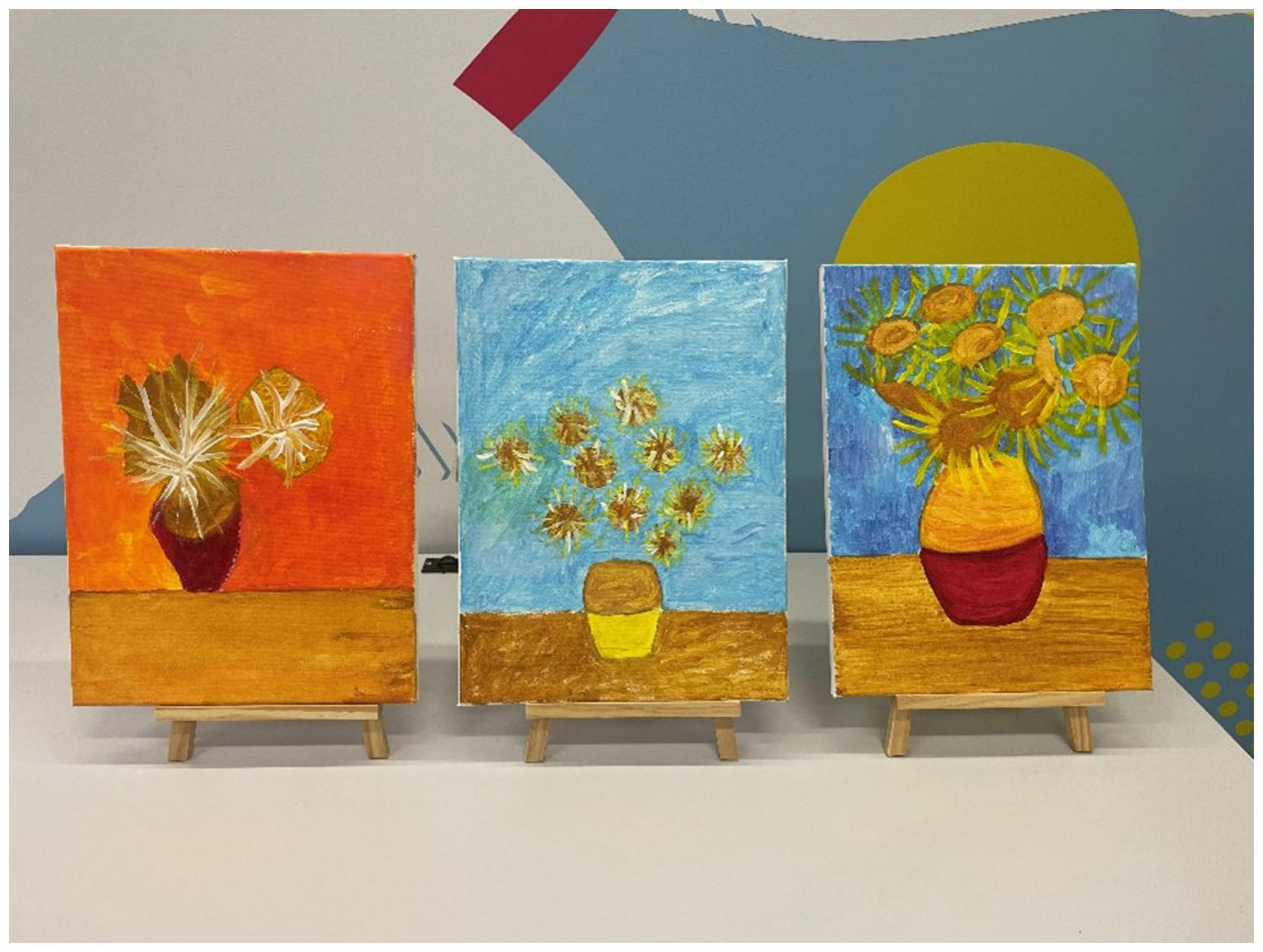
An example of paintings from an art therapy session.

Interventions consisted of the following face-to-face sessions at the clinic:

- Group and individual cognitive stimulation delivered by a NP and registered nurse (RN) (60–120 min, weekly), using activities such as puzzles, LEGO® and games (Figure 1).
- Group and individual art therapy delivered by a NP and RN (60 min, weekly), using paint, clay, craft and drawing materials (Figure 2).
- Group and individual exercise interventions delivered by an exercise physiologist (30–60 min, weekly). The sessions ranged from low to medium intensity, and included body weight exercises, treadmill, exercise bike and resistance training.
- Low intensity group yoga delivered by a trained yoga instructor and RN (30 min, every fortnight).
- Participant requested appointments with the NP to discuss health and wellbeing issues and facilitate referrals to other services and healthcare practitioners (such as Dementia Australia) (30–90 min).

### Participants

Participants were eligible if they were aged ≥18 years, had a clinical-confirmed diagnosis of dementia, lived in the community (not residential aged care), able to verbalise and understand English, able to travel to the clinic, and able to provide written informed consent or a substitute decision maker that can provide consent on their behalf. Recruitment occurred between September 2022 and June 2023 using convenience sampling with participants referred to the clinic by healthcare practitioners. The healthcare practitioners who referred people to the clinic were geriatricians, exercise physiologists, registered nurses, and occupational therapists.

### Data collection

Demographics were collected, including age, gender, dementia type, living situation, current support services, and driving status. Attendance at the clinic was recorded. Approximately every 6 months over a 12-month period, participants were invited to a clinical interview (total of 60 min each). Baseline data were collected during the initial appointment with the NP. Details such as medical history, social history (e.g., hobbies, occupation), and concerns around their dementia diagnosis and goals were recorded in clinical notes.

Cognitive and psychological profiles were assessed using validated screening and assessment tools (Table 1) to determine changes in *cognition* [Rowland Universal Dementia Assessment Scale (RUDAS)] (Storey et al., 2004), *psychological wellbeing* [Depression Anxiety Stress Scale (DASS-21)] (Gates et al., 2014), and *quality of life* [Quality of Life in Alzheimer’s Disease (QOL-AD)] (Thorgrimsen et al., 2003).

**Table 1:** Clinical interview validated tools for the person with dementia.

Psychometric measure	Description
Rowland Universal Dementia Assessment Scale (RUDAS)	Six item clinician-administered scale that assesses multiple cognitive domains including memory, praxis, language, judgement, drawing and body orientation. Scores of 23 or less indicate cognitive impairment (Storey et al., 2004).
Depression Anxiety Stress Scale (DASS-21)	A self-report scale designed to measure the negative emotional states of depression, anxiety and stress. It consists of 21 items, with each item rates on a 4-point Likert scale from 0 (never) to 3 (almost always). The scale is divided into three 7-item subscales for Depression, Anxiety, and Stress. To calculate the final score of each subscale, the total raw score (sum of the 7 items) is multiplied by 2. Higher scores indicate greater severity of symptoms. Severity ratings for each subscale are classified as normal, mild, moderate, severe and extremely severe based on established cut-off scores (e.g., Depression: 0–9 normal, 10–13 mild, 14–20 moderate, 21–27 severe, ≥28 extremely severe; Anxiety: 0–7 normal, 8–9 mild, 10–14 moderate, 15–19 severe, ≥20 extremely severe; Stress: 0–14 normal, 15–18 mild, 19–25 moderate, 26–33 severe, ≥34 extremely severe) (Gates et al., 2014).
Quality of Life in Alzheimer’s Disease (QOL-AD)	Thirteen item clinician-administered scale assessing various domains of quality of life, such as physical and mental health for people with dementia. Each question is rated on a Likert scale of 1 (poor) to 4 (excellent). The total score ranges from 13 (lowest quality of life) to 52 (highest quality of life) (Thorgrimsen et al., 2003). Scores are interpreted on a continuum, with no standardised severity cut-offs; instead, differences in scores are used to reflect relative levels of quality of life and change over time.

### Data analysis

Data were analysed using IBM SPSS 28.0. Descriptive statistics were used, and variables were described using frequency, mean, and standard deviation (SD). Due to the small sample size and the heterogeneous nature of dementia, participant data are reported individually. Outcome measures were evaluated descriptively by examining within-participant change across timepoints (baseline, 6 months and 12 months), with baseline values used as the reference point for assessing change over time. No comparator group was included. Given the small sample size and exploratory nature of this pilot, no imputation or formal missing data procedures were undertaken. Presentation of data was restricted to complete cases, with results reported for participants who had data available at baseline, 6-months, and 12-months timepoints to support consistent longitudinal interpretation. As this was not a pilot study of efficacy, the size of effect or power analysis is not presented. Purposeful sampling was undertaken to select a sub-sample of participants to capture brief vignettes of people who attended the clinic. Data from the observations and clinical notes were used to complement the quantitative outcome measures.

### Ethical considerations

Human Research Ethics approval for the conduct of this study was gained from the University Wollongong (HREC) prior to commencing recruitment and data collection (Approval No 2022/082). In order to respect the autonomy of the people with dementia, we sought written consent or assent from them. We used process consent as described by Dewing (2008). In the event that the person with dementia was assenting but was unable to provide consent, consent was obtained from their substitute decision maker. Written consent was obtained following the initial appointment with the NP.

## Results

A total of 16 people with dementia attended the clinic between September 2022 and June 2023, and eight participated in the study (recruitment rate 50.0%). Of these, six participants attended the clinic for 12-month period (completion rate 70.0%). Table 2 shows the demographic information for consenting participants (*n =* 8). Participants attending the clinic ranged from 3 months to 5 years post dementia diagnosis.

**Table 2:** Baseline demographics of the people with dementia.

	*n* (%)	*M* (range)
Gender		
Female	3 (37.5)	
Male	5 (62.5)	
Age		77.5 (63–91)
Type of dementia		
AD	3 (37.5)	
Mixed	1 (12.5)	
Vascular	2 (25)	
Younger onset AD	1 (12.5)	
Unknown	1 (12.5)	
Who is the person with dementia living with?		
Living with spouse	6 (75.0)	
Living with their child	1 (12.5)	
Living alone	1 (12.5)	
Does the person with dementia have support services in place (i.e., home care package)?		
Yes	3 (37.5)	
No	5 (62.5)	
Is the person with dementia driving?		
Yes	3 (37.5)	
No	5 (62.5)	
Who was consent provided by?		
By the person with dementia	6 (75.0)	
By the substitute decision maker	2 (25.0)	

Of the six participants who attend the clinic for 12 months, data at baseline, 6-months and 12-months were available for four participants as presented in Table 3. Data were not available for two participants at one or more of the timepoints due to participant unavailability and, in some cases, inability to complete data collection within the scheduled timeframe due to staffing constraints.

**Table 3:** Participant results.

Participant pseudonym	Time point	QoL-AD score	RUDAS score	DASS-21 depression score	DASS-21 anxiety score	DASS-21 stress score	Total number of weeks attended	Types of sessions attended
Nora (Female, 63)	T0 (Baseline)	34	Declined to be assessed	2	0	2	45	Art therapy, cognitive stimulation and exercise sessions
	T2 (6 months)	35	Declined to be assessed	1	0	1		
	T4 (12 months)	34	Declined to be assessed	2	4 (mild anxiety)	2		
Frank (Male, 78)	T0 (Baseline)	39	28	8 (moderate depression)	5 (mild anxiety)	4	43	Cognitive stimulation and exercise sessions
	T2 (6 months)	39	29	4	5 (mild anxiety)	0		
	T4 (12 months)	32	30	4	4 (mild anxiety)	3		
Lloyd (Male, 72)	T0 (Baseline)	47	20 (cognitive impairment)	0	0	0	29	Cognitive stimulation and exercise sessions
	T2 (6 months)	46	21 (cognitive impairment)	0	0	0		
	T4 (12 months)	47	19 (cognitive impairment)	1	4 (mild anxiety)	4		
Harvey (Male, 76)	T0 (Baseline)	50	30	0	1	1	34	Yoga, art therapy, cognitive stimulation and exercise sessions
	T2 (6 months)	52	30	3	3	0		
	T4 (12 months)	48	28	0	0	1		

QoL-AD, Quality of Life Alzheimer’s Disease; RUDAS, Rowland Universal Dementia Assessment Scale; DASS-21, Depression Anxiety Stress Scale (21-item).

## Case vignettes

### Nora (participant 0001)

Prior to her diagnosis of dementia, Nora, had been an artist. However, an injury to her right hand and visual spatial changes from dementia, were impacting her ability to engage in painting. Nora was also having minor falls and difficulty using stairs. Nora wanted to be able to continue to walk her dog by the beach each day. Despite scoring 0 for anxiety on the DASS-21 (Table 3), Nora expressed feeling anxious. Nora selected to participate in art therapy (involving painting and clay work) and individual exercise physiology. After 10 months, Nora, asked to work on a jigsaw puzzle instead of art. Nora was provided with 1:1 support to enable her to achieve her goal of completing a 500-piece puzzle. Nora was able to continue to walk her dog each day. In line with the stable quantitative outcomes shown in Table 3, Nora maintained functional participation over time. However, her reported anxiety despite a DASS-21 score of 0 highlights a divergence between standardised measures and lived experience.

### Frank (participant 0002)

Frank lived an active lifestyle, going for 5 km walks up to four times a week with friends. However, Frank reported having trouble keeping up with the social conversation and speed of the walk. Frank spent 2 days a week maintaining historical trains but expressed starting to have difficulty with fine motor skills. Frank wanted to be able to continue socialising, walking with his friends, and maintaining historical trains. Frank selected to participate in group exercise physiology and cognitive stimulation (specifically LEGO®). Frank initially needed 1:1 support to complete simple LEGO® kits, however, within 1 month he was completing complex LEGO® kits with minimal support. Over the 12-month period, Frank completed 10 LEGO® kits and continued socialising, walking and train maintenance. While Table 3 reflects overall stability in quantitative outcomes, Frank’s progression from requiring 1:1 support to independently completing complex tasks illustrates how stable scores may mask meaningful functional gains in confidence and participation.

### Lloyd (participant 0004)

Lloyd was a keen golfer and had been a builder. Lloyd expressed concerns in losing his ability to play golf and to drive his car. Lloyd selected to participate in group exercise physiology and cognitive stimulation. Lloyd participated in a range of cognitive stimulation activities- LEGO®, puzzles (e.g., math, find-a-word), jigsaw puzzles, games (e.g., memory, quoits, checkers, cards, dominos, Jenga). Lloyd selected different activities each week and would often complete several activities a session. Some sessions, Llyod would choose to make a coffee and have a social chat. Lloyd continued playing golf and driving over the 12-month period. This sustained engagement and continuation of valued activities are consistent with the stability observed in Table 3, suggesting maintenance of functional ability and participation over time despite the progressive nature of dementia.

### Harvey (participant 0008)

Harvey was a mechanical engineer, rower, gardener, and ballroom dancer. Harvey’s goal was to maintain being active and creative. Harvey selected to participate in exercise physiology, art therapy, cognitive stimulation, and yoga. While Harvey predominantly focused on LEGO® each week, he also undertook other activities such as art therapy and puzzles. Eight months after joining the clinic, Harvey experienced a delirium following a trip overseas resulting in functional and cognitive decline, and his driver’s licence was temporarily suspended. Harvey recovered from delirium and his driver’s licence was reinstated. Despite this acute clinical event, Harvey’s outcomes remained stable in Table 3, suggesting maintenance of function over time. This case highlights that standardised measures may not fully capture short-term disruptions, while illustrating the potential for recovery and overall functional preservation within the program.

## Discussion

This brief report presented the design, intervention development, and preliminary outcomes of an NP-led post-diagnostic clinic for people with dementia. While based on a small sample size, the findings suggest that participants maintained their cognition, psychological wellbeing, and quality of life over the 12-month follow-up period, highlighting the clinic’s potential role in supporting stability in these domains.

Maintaining cognition, psychological wellbeing, and quality of life is crucial for people with dementia due to the progressive nature of the disease, and declines in these domains can impact daily functions, independence, and overall wellbeing. There is shifting focus from a fatalistic perspective on dementia to a more empowering narrative that seeks to support people with dementia to live well (Quinn et al., 2022). Living well with dementia is highly individualised, and a one size fits all approach is outdated. The strength of the NP-led post-diagnostic clinic model is the unique and individualised way in which people with dementia were enabled to have choice in the activities and sessions they engaged with, reflecting person-centred values. Having choice is crucial for people with dementia to contribute to a sense of identity and living well with the condition (Quinn et al., 2022). While choice as a concept was not formally captured within the findings, the premise of the program design was to facilitate choice of activities with participants. Future studies could explore how enabling choice impacts engagement, wellbeing, and outcomes in similar models of care.

The clinic builds on existing models of NP-led clinics for people with dementia (Luck & Doucet, 2021) with the addition of non-pharmacological treatments aimed at having a positive impact on cognition, psychological wellbeing, physical function, and quality of life for people with dementia. A NP-led mobile memory clinic has been evaluated in Australia with high levels of acceptability and satisfaction for people with dementia and care partners (Pond et al., 2021). However, the financial viability and lack of NPs were barriers to expansion in the community care setting. Non-pharmacological treatments have the potential to be replicated and embedded into existing memory clinics or outpatient rehabilitation clinics, as demonstrated in a 12-week multicomponent program in the Australian Capital Territory, Australia (D’Cunha et al., 2025).

### Limitations of the work

Due to this being a clinician-led and unfunded study there were difficulties in data collection. Priority was given to the clinical intervention rather than data collection. Participant absences due to travel and short-term illness contributed to difficulties in data collection. The limited sample size is attributable to the clinic’s restricted operating schedule, as it was open for only 1 day each week. Additionally, the quantitative data does not capture the lived experience and subjective perspectives of the people with dementia, including their perceptions of the benefits and impact of attending the clinic.

### Implications for policy and practice

Funding is needed to further support the development, implementation and research of specialist post-diagnostic clinics that offer non-pharmacological treatments for people with dementia. Currently, in Australia funding is focussed on medications, with non-pharmacological and multidisciplinary treatments for people with dementia overlooked (Pavković et al., 2025). In 2025, the Australian Government introduced the Support at Home program which includes Restorative Care Pathway, aiming to increase strength and capabilities through allied health services for up to 16 weeks (Australian Government Department of Health and Aged Care, 2025). At present, details of the new funding model relating to access to centre- and group-based programs are unclear, and it remains to be seen whether expansion of post-diagnostic clinic models will be feasible. Scaling up of non-pharmacological treatments, such as CST (Knapp et al., 2022) and the Care of People with dementia in their Environment (COPE) program (Rahja et al., 2020) have been shown to offer economic benefits. Therefore, investment in post-diagnostic support programs for people with dementia is required to reduce costs to the health care system resulting from hospitalisations, falls, and care partner exhaustion.

## Conclusion

This brief report provides foundational work and structural guidance to develop and implement person-centred non-pharmacological NP-led models of post-diagnostic care for people with dementia. Findings from this small exploratory sample suggest that such models are feasible and may support engagement in tailored, non-pharmacological interventions aligned with individual goals. Post-diagnostic care should be designed to enable the person to have choice to enhance meaningful engagement in support of their goals.

Given the small sample size, findings should be interpreted cautiously. Further research with larger samples, including feasibility studies and randomised controlled trials, is needed to evaluate the effectiveness, scalability and economic impact of NP-led post-diagnostic clinic models incorporating non-pharmacological interventions for people with dementia. This study has informed a subsequent qualitative investigation exploring participants’ experiences of the clinic and its impact on living well with dementia.

## Data Availability

The data supporting the findings of this study are available upon reasonable request from the corresponding author.
